# A Critical Evaluation of the Gamma‐Hydroxybutyrate (GHB) Model of Absence Seizures

**DOI:** 10.1111/cns.12337

**Published:** 2014-11-18

**Authors:** Marcello Venzi, Giuseppe Di Giovanni, Vincenzo Crunelli

**Affiliations:** ^1^ Neuroscience Division School of Bioscience Cardiff University Cardiff UK; ^2^ Department of Physiology and Biochemistry Malta University Msida, Malta

**Keywords:** Cortex, EEG, GABA‐B receptors, Hypnosis, Sedation, Thalamus

## Abstract

Typical absence seizures (ASs) are nonconvulsive epileptic events which are commonly observed in pediatric and juvenile epilepsies and may be present in adults suffering from other idiopathic generalized epilepsies. Our understanding of the pathophysiological mechanisms of ASs has been greatly advanced by the availability of genetic and pharmacological models, in particular the *γ*‐hydroxybutyrate (GHB) model which, in recent years, has been extensively used in studies in transgenic mice. GHB is an endogenous brain molecule that upon administration to various species, including humans, induces not only ASs but also a state of sedation/hypnosis. Analysis of the available data clearly indicates that only in the rat does there exist a set of GHB‐elicited behavioral and EEG events that can be confidently classified as ASs. Other GHB activities, particularly in mice, appear to be mostly of a sedative/hypnotic nature: thus, their relevance to ASs requires further investigation. At the molecular level, GHB acts as a weak GABA‐B agonist, while the existence of a GHB receptor remains elusive. The pre‐ and postsynaptic actions underlying GHB‐elicited ASs have been thoroughly elucidated in thalamus, but little is known about the cellular/network effects of GHB in neocortex, the other brain region involved in the generation of ASs.

## Introduction

**Figure 1:**
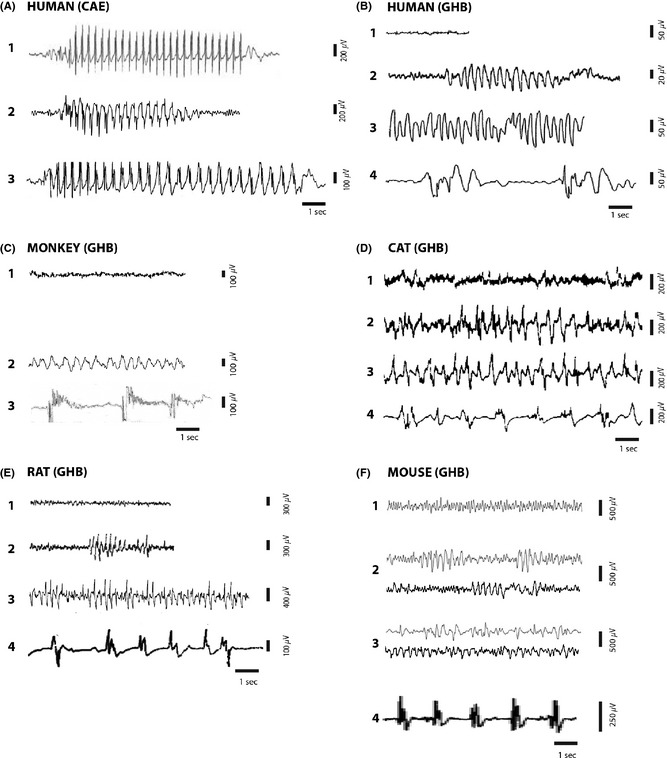
EEG recordings of human ASs and EEG activities elicited by GHB in various animal species. (**A**) Scalp EEG recordings from three childhood absence epilepsy (CAE) patients showing the characteristic 3–4 Hz SWDs, the EEG hallmark of ASs. Note the sudden onset and termination of the SWDs from a desynchronized EEG background, and the different SWD morphology among patients (i.e., different amplitude of the spike component both within a SWD (2,3) and in different patients (1–3). (**B**) Administration of GBL (30 mg/kg i.v.) to healthy human volunteers produces 2–3 Hz delta waves (2) that appear suddenly from a desynchronized EEG background (1). After 10–15 min, the delta waves become continuous (3). This EEG output can be obtained for a range of GBL and GHB doses (3–6 g GHB i.v.). Doses exceeding 7–8 g i.v. produce, following the EEG manifestations shown in (2) and (3), a burst‐suppression pattern (4) where bursts of slow EEG waves interrupt cortical silence. This EEG activity is invariably accompanied by a state of deep hypnosis/anesthesia. (see Figure 2 for a comparison of the effect of GHB in a patient with generalized epilepsy.) (**C**) In monkeys, the desynchronized EEG (1) evolves into an EEG pattern of 3 Hz slow/delta waves following administration of 200 mg/kg/s.c. of GBL (2). High doses of GHB (500 mg/kg/i.v.) produce, in addition to the EEG manifestations shown in (2), a clear burst‐suppression pattern (3). (**D**) In cats, an i.p. injection of GHB 200 mg/kg induces first an intermittent (2) and then a continuous hypersynchronous EEG (3). The EEG is punctuated with spikes that are found either alone or within 2–3 Hz SWCs. GHB 400 mg/kg i.p. induces, in addition to the EEG manifestations shown in (2) and (3), a burst‐suppression pattern (4). (**E**) In rats, a dose of 200 mg/kg GHB i.p. elicits at first isolated 5–6 Hz SWDs (2) that emerge from a desynchronized EEG background (1). Within 10–15 min, this EEG activity becomes continuous and its frequency slows down to 4–5 Hz (3). Note that SWCs are not always discernible, and slow waves without spikes are sometimes prevalent. A dose of 400 mg/kg GBL induces, subsequently to the EEG manifestations shown in (2) and (3), a burst‐suppression pattern (4). (**F**) In mice, a dose of 70 mg/kg GBL i.p. induces first the appearance of 4–5 Hz SWDs (2, top trace) or 4–5 Hz waves (2, bottom trace) that appear intermittently in the EEG. This EEG activity gradually becomes continuous and its frequency slows down (3), still exhibiting spike and waves (top trace) or waves only (bottom trace). A dose of 150 mg/kg GBL elicits, following the EEG manifestations shown in (2) and (3), a burst‐suppression pattern (4). Reproduced (with and without modification) from 2 A1; 160 A2; 14 A3; 32 B1‐2‐4; 47 B3; 54 C1‐2; 51 C3; 33 D; 58 E1‐2‐3; 64 E4; 155 F1, F2‐3 (top); 69 F2‐3 (bottom); 70 F4.

Typical absence seizures (ASs) are brief (3–30 second) nonconvulsive epileptic events that consist of impairment of consciousness accompanied in the electroencephalogram (EEG) by 2.5–4 Hz “spike and slow‐wave discharges” (SWDs) (Figure 1A) 1. ASs start and end abruptly and there is no aura or postictal depression 1, 2. The extent of the impairment of consciousness is variable among individuals, and between seizures in the same individual, and is generally defined by a lack of responsiveness to external stimuli during the seizure, a temporary interruption of an ongoing task (although simple repetitive tasks can continue during ASs) and/or the inability to recall, after seizure termination, a stimulus that had occurred ictally 2, 3. Although ASs are part of a more complex phenotype in many idiopathic generalized epilepsies, they are the only clinical symptom in childhood absence epilepsy (CAE), a common pediatric epilepsy, which accounts for about 10% of all childhood epilepsies 4, 5, 6. CAE generally affects children between 4 and 10 years, has a remission rate of between 20% and 70% 1, 7, and a clear polygenic inheritance 1, 8, 9.

The current pharmacological treatment of ASs is based on “classical” antiabsence drugs, namely ethosuximide and valproate, each effective in about 50% of patients 10. Both drugs have recently been shown to be more efficacious than newly developed drugs, such as lamotrigine 10. In addition, antiepileptic drugs that are effective against convulsive seizures (such as carbamazepine and phenytoin) are generally reported to be either ineffective or to aggravate human ASs 11, 12, 13, 14, making the pharmacological profile of ASs unique.

The pathophysiological mechanisms of typical ASs are only partly understood, but it is well established that ASs are generated by abnormal electrical activity in reciprocally connected thalamic and cortical territories, that is, the thalamocortical (TC) network 1, 15. Indeed, the integrity of all of the main components of the TC network, that is, thalamic nuclei, the nucleus reticularis thalami (NRT), and the cortex, is essential for the full expression of the behavioral and EEG features of experimental ASs 1, 16. Imaging studies in humans have shown that the cerebellum and limbic structures (such as the hippocampus) are not involved in the expression of typical ASs 17, 18, 19, although they may play a role in atypical ASs 20.

Various genetic and pharmacological animal models of ASs have been developed to help understand the mechanisms underlying the generation of these nonconvulsive seizures. Two polygenic rat strains, namely GAERS (genetic absence epilepsy rats from Strasbourg 21) and WAG/Rij (Wistar Albino Glaxo rats from Rijswijk 22) are the best‐characterized genetic models of ASs consisting of 7–9 Hz SWDs and concomitant behavioral arrest in the absence of other neurological abnormalities. In addition, several monogenic mouse models of ASs have been described (e.g., stargazer, lethargic, tottering, etc. 1, 23, 24, although these models also present additional neurological phenotypes such as ataxia.

Pharmacological models of ASs have also been developed. Historically, *γ*‐hydroxybutyric acid (GHB), pentylenetetrazol (PTZ) (at low doses, 20–30 mg/kg), and penicillin (at high doses, intramuscularly) have been the drugs most commonly used to induce ASs in various animal species 25. Although, undoubtedly, genetic models of ASs have many advantages, both in theoretical (i.e., construct validity) and in practical terms, the use of pharmacological models, and in particular the GHB model, has increased in recent years. One of the reasons is that AS‐inducing drugs can be applied systemically to transgenic mice to investigate the contribution of individual genes to the expression of ASs. Crossing a genetic mouse model of ASs with a transgenic mouse would also be a viable alternative, but it is more time consuming because, to control for differences in genetic backgrounds, the resulting strain needs to be backcrossed with the recipient strain for several generations 24, 26. Moreover, using a substance capable of inducing ASs in various species, it is possible to combine a broad range of invasive and noninvasive techniques, pharmacological interventions, and behavioral paradigms to allow a more comprehensive definition of both the face and the predictive validity of an AS model.

This review provides a critical evaluation of the GHB model of ASs and its relevance to the human condition. We start by describing the EEG and behavioral correlates of exogenous GHB administration in humans and compare them to those in nonhuman primates, cats, and rodents. We then review the brain regions involved in the expression of GHB‐elicited ASs and the molecular targets of the GHB in the brain. What emerges from this analysis is that only in the rat does there exist a subset of GHB‐elicited behavioral and EEG events that can be confidently classified as ASs. In other species, further studies are required to assess the potential relevance of other GHB‐induced activities (i.e., sedation/hypnosis) to ASs. At the molecular level, the thalamic GABA‐B receptor‐mediated pre‐ and postsynaptic actions that underlie GHB‐elicited ASs have been elucidated, while its cellular and network effects in the neocortex are still largely unknown.

## Brief History of GHB

GHB was originally synthetized, starting from *γ*‐butyrolactone (GBL), by Henri Laborit in 1960 in an attempt to create a novel anesthetic agent that would cross the blood‐brain barrier and act as a GABA analog 27. In humans, however, GHB was never used as an anesthetic agent in isolation; although it initially gained some popularity as a sedative/hypnotic agent, or as an adjuvant to other anesthetics 28, 29. It was subsequently discovered that GHB is actually an endogenous brain substance, raising the possibility of a role for endogenous GHB in natural sleep 30. In subsequent years, the pathways which control the brain levels of GHB and its conversion to GABA were also described 31.

Notwithstanding initial claims that GHB had some antiepileptic properties 27; anecdotal evidence suggested that GHB could induce ASs in subjects with a history of generalized seizures 32. Nonetheless, it was animal studies that first indicated that the exogenous application of GHB induces a state that bore more resemblance to ASs than to anesthesia/sleep. Historically, the first evidence of the ability of GHB to elicit nonconvulsive, generalized seizures in a naïve animal came from a study in the cat 33. This finding was partially supported by studies in monkeys 34 and rats 35, but it was mostly the work of Carter Snead's group that established GHB as a solid model of ASs 25, 36, 37.

Importantly, it was later established that GBL, which is per se biologically inactive 34, 38, 39, can be converted into GHB by a lactonase in the plasma and liver, and therefore acts as a GHB prodrug 40. GBL has now supplanted GHB for the induction of experimental ASs because of its faster onset of action 41, but, given that GHB is the active form of the drug, for clarity, we will thereafter continue referring to the “GHB model” or “GHB‐elicited” activity, even if the administered drug was actually GBL.

For the sake of completeness, it should be noted that chronic GHB administration has found some therapeutic applications in humans, for instance in the treatment of narcolepsy and alcohol‐withdrawal syndrome. Moreover, the abuse potential of GHB is becoming increasingly recognized. These aspects of therapeutic use and abuse of GHB will not be described here, and the reader is referred to relevant reviews on the subject 42, 43, 44.

## EEG and Behavior Following Acute GHB Administration: Hypnotic and Seizure‐Like Activities

### Species‐Specific Effects of GHB Administration

GHB has been characterized as a model of ASs across various species for more than 50 years. Given that the terminology used to describe the EEG and behavioral effects of GHB varies markedly across studies, we will describe the effects of GHB administration in various animal species, while being faithful to the original terminology used in each of the original reports (Table 1). In view of the peculiar pharmacological profile of human ASs 25, special attention will be given to the sensitivity of the various GHB‐elicited activities to antiepileptic drugs (Table 2).

**Table 1:** EEG activities evoked by GHB in different species

Species	Drug and dose	Route	Stage2a		Stage2b		References
			Frequency	Description	Frequency	Description	
**Human**							
Human	GHB 3–6 g	i.v.	?	?	2–3 Hz	Monomorphic delta waves	32
Human	GBL 25–30 mg/Kg	i.v.	2–5 Hz	Slow waves	2–2.5 Hz	Slow waves	47
Non‐human primate							
Rhesus monkey	GHB 200–400 mg/kg	i.v.	?	?	2.5–3 Hz	High‐voltage slow waves often associated with spikes (note spikes are not visible in the figures)	51
Marmoset monkey	GBL 200 mg/Kg	s.c.	?	?	3 Hz	SWDs with spikes that are not discernible	54
**Cat**							
Cat	200–400 mg/Kg	i.p.	2–3 Hz	Intermittent hypersynchronous bursts	2.5 Hz	Continuous hypersynchronous waves composed of one of three complexes, that is, slow waves, a slow wave followed by a spike or a slow wave followed by a short polyphasic burst discharge.	33
**Rat**							
Sprague‐Dawley	500 mg/Kg GHL/700 mg/Kg GHB	i.p.	?	Intermittent hypersynchronous waves	2–3 Hz	Continuous hypersynchrony	35
Sprague‐Dawley	400 mg/Kg GBL	i.p.	?	Brief bursts of spikes	?	Continuous spiking and/or spike and slow wave	64
Sprague‐Dawley	100 mg/Kg GBL	i.p.	4–6 Hz	SWDs	?	Continuous SWDs	124
Sprague‐Dawley	100 mg/Kg GBL	i.p.	5–6 Hz	Bursts of spikes	?	Continuous spiking	148
Sprague‐Dawley	150 mg/Kg GBL	i.p.	7–9 Hz	SWDs	?	?	25
Sprague‐Dawley	100 mgkg GHB	i.p.	6–9 Hz	SWDs	?	?	62
Sprague‐Dawley	200 mg/Kg GBL	i.p.	6–7 Hz	SWDs	?	?	87
Wistar	200 mg/Kg GHB	i.p.	5–6 Hz	Bursts of hypersynchronous waves	4–5 Hz	Continuous hypersynchrony	58, 59
Wistar	100 mg/Kg GBL	i.p.	4–5 Hz	SWDs	?		158
Wistar	200 mg/Kg GBL	i.p.	4–5 Hz	SWDs	?	Continuous SWDs	158
**Mouse**							
Ddy	100 mg/Kg GHB or GBL	i.p.	3–6 Hz	SWDs	3–6 Hz	SWDs	70
C57BL/6J	70 mg/Kg GBL	i.p.	3–5 Hz	SWDs	3–5 Hz	SWDs	69
BALB/cJ	100–150 mg/Kg GBL	i.p.	?	Burst of hypersynchronous slow waves	?	Hypersynchronous slow waves and/or spiky EEG	68
C57BL/6	100–150 mg/Kg GBL	i.p.	?	Hypersynchronous slow waves and/or SWD	?	Hypersynchronous slow waves and/or SWD	157

The description of stage 2a and 2b reports the wording used in the original papers. For further details of the classification of stage 2a and 2b see main text. i.p. intraperitoneal; i.v. intravenous; s.c. subcutaneous; NA/?: data not available.

**Table 2:** Comparison of the pharmacological profile of human ASs and GHB‐elicited stage 2 activities (ASs and hypnosis). For further details of the classification of stage 2 activities into hypnosis and ASs see section 3.2

	Antiabsence drugs			Drugs ineffective or worsening ASs		References
	Ethosuximide	Valproate	Lamotrigine	Carbamazepine	Phenytoin	
Human ASs (CAE)	↓	↓	↓	↓/=	↑/=	10, 11, 12, 13, 14
GHB‐elicited stage 2 activities						
Human	?	?	?	?	?	NA
Monkey	↓	?	?	?	↑/=	52, 53, 54
Cat	?	?	?	?	?	NA
Rat	↓	↓	?	↑	↑	25, 59, 62, 63
Mouse	↓	=	?	?	?	70

↓: decrease of ASs; ↑: exacerbation of ASs; =: no effect on ASs; NA/?: data not available.

#### Humans

Early reports described the effect of GHB, administered intravenously (i.v.) in doses of 3–10 g (~40–140 mg/kg), on the EEG and behavior of healthy volunteers 27, 32. These early experiments are of particular interest because they show effects of GHB at higher doses than those currently used therapeutically.

Sedation appeared within 5–10 min from the beginning of the administration of the drug 32, 45, 46. This was accompanied by the disappearance of the alpha rhythm in the EEG along with an increase in theta activity, without any apparent change in behavior 32. This stage was followed by the occurrence of high‐amplitude delta waves in the EEG (Figure 1B3), while the subject appeared to be drowsy. At a dose of 3 g i.v., the subjects descended into a state of reversible sleep, but still responded to sensory stimulation which produced a temporary disappearance of the delta waves and EEG desynchronization for the duration of the stimulus. Furthermore, the subjects had difficulty in performing mental calculations, pointing to a disruption of cognitive function 32. Another study using GBL (20–30 mg/kg i.v.) also produced 2–5 Hz slow waves, which appeared initially as intermittent bursts (Figure 1B2) and then became continuous within 15 min of the injection 47. Interestingly, the author of this study claims that consciousness was spared in these subjects, that is, although the subjects felt mildly intoxicated, they were aware of their surroundings and could perform tasks such as counting light flashes. During this behavioral output, the slow waves were replaced by a desynchronized EEG 47. At doses of 4–5 g, the sensory threshold to awaken a subject who was in the delta wave stage was higher, and only painful stimulations could produce a desynchronized EEG and a behavioral response (e.g., movement). With doses of 7–8 g of GHB, the appearance of delta waves was followed by another characteristic stage: the EEG displayed cortical silence, interrupted by K‐complexes (Figure 1B4), while behaviorally, the subject was unresponsive to external stimuli, including nociceptive ones, that is, the subject was anesthetized 32. This EEG manifestation, called “burst‐suppression pattern”, is also characteristic of the anesthetic state induced by thiopental, propofol, and isoflurane 48, 49, 50. In summary, in healthy volunteers, there is no evidence that GHB induces SWDs or ASs, and, importantly, no antiabsence drug has been tested against the GHB‐elicited slow/delta waves, burst‐suppression pattern, and respective behaviors that are elicited by GHB.

**Figure 2:**
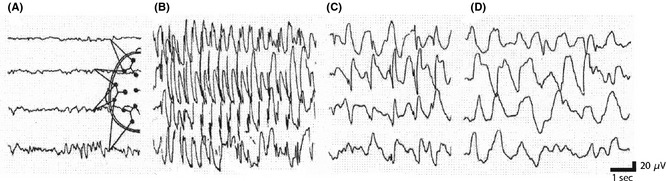
GHB administration induces SWDs in subjects with generalized epilepsy. (**A**) EEG from a patient with generalized epilepsy prior to GHB administration. Bolus i.v. injection of GHB (3 g) induced SWDs in the EEG within 2 min from the injection (**B**). SWDs were of short duration and gave rise to slow/delta waves (**C**), initially punctuated with spikes (compare with Figure 1B). The EEG spikes gradually disappeared from the slow/delta waves background (**D**) so that the EEG became similar to the one reported for GHB administration in healthy subjects (compare with Figure 1B). Modified from 32.

However, GHB has been shown to have a pro‐epileptic effect in patients with a history of (nonidentified) generalized seizures 32. Indeed, in these patients, SWDs were observed in the EEG within 2 min of an i.v. bolus injection of 3 g of GHB (Figure 2B). These SWDs, however, were short lived: within few minutes, the spikes started to slowly disappear and the frequency of the EEG large amplitude waves became progressively slower (Figure 2C), eventually giving rise to full‐blown delta waves (Figure 2D) similar to those observed after administration of an equivalent dose of GHB to healthy subjects (see Figure 1B3). Unfortunately, no description of the behavioral correlates (e.g., impairment of consciousness) that accompanied the EEG expression of SWDs was provided and no antiabsence drug was tested against the GHB‐elicited SWDs 32.

#### Nonhuman Primates

I.v. administration of GHB in nonhuman primates elicited similar patterns of EEG activity to those seen in healthy humans (Figure 1C). In rhesus monkeys, a low dose of GHB (100–200 mg/kg) induced low‐voltage slowing of the EEG, accompanied by drowsiness 51. At a dose of 400 mg/kg 52, a continuous activity, characterized by 2–3 Hz high‐voltage slow waves, appeared in the EEG. Animals were unresponsive to sensory stimulation and displayed occasional stereotyped movements and myoclonic jerks. At even higher doses (>500 mg/kg), animals started to display generalized myoclonic jerks accompanied by a burst‐suppression EEG pattern (Figure 1C3) 51. These EEG and behavioral effects were blocked by ethosuximide, given both acutely (100 mg/kg i.v.) and chronically (serum concentration: 140 *μ*g/mL), and were worsened by chronic treatment with phenytoin (serum concentration: 14 *μ*g/mL) (Table 2) 52, 53. These results have recently been replicated in marmoset monkeys with i.v. injection of 200 mg/kg GBL producing a similar slow‐wave EEG pattern (Figure 1C2) and associated behavior, both of which were reversed by chronic treatment with ethosuximide (30 mg/kg/day) 54.

On the basis of the co‐occurrence, and unique pharmacological profiles, of the behavioral output and EEG paroxysm, it was argued that the GHB‐elicited activity in monkeys modeled the spontaneous ASs of idiopathic generalized epilepsy. However, as in healthy humans treated with GHB, the presence of spikes superimposed to the slow/delta waves is not discernible in the EEG recordings of GHB‐treated monkeys, and in contrast to spontaneous ASs in humans, the slow/delta wave activity could be evoked by auditory stimulation 51.

#### Cats

Injection of GHB, either i.v. or intraperitoneally (i.p.), produced EEG and behavioral changes similar to those that have been described in primates 33, 55, 56, with some important differences: no drowsiness was observed at low doses (e.g., 60 mg/kg), and the EEG slowing was accompanied by the presence of spikes in the EEG 33. Upon i.p. administration of 200–400 mg/kg of GHB, the animal first produced an EEG pattern defined as “2–3 Hz intermittent hypersynchronous bursts” (Figure 1D2) 33, while it was in a crouching position with its eyes open. This gradually progressed into a pattern of “2.5 Hz continuous hypersynchrony, where each complex is composed of either a slow wave, a slow wave followed by a spike or a slow wave followed by a polyphasic burst discharge” (Figure 1D3) 33. During this EEG state, the animal had fixed gaze and made repetitive head movements. Sensory stimulation disrupted the EEG synchrony and awakened the cat. Following a dose of 400–600 mg/kg, the EEG progressed through the previously described continuous and intermittent EEG stages and then showed a burst‐suppression pattern (Figure 1D4) that was accompanied by myoclonic jerks 33. No antiabsence drugs were tested against the EEG and behavioral phenotype elicited by GHB in cats.

#### Rats

The effects of GHB in rats are by far the best described among all species. Systemic administration of GHB (25–100 mg/kg) in Wistar rats produced an increase in slow‐wave sleep 57, 58 that persisted for up to 4 h. Higher doses (200 mg/kg) in Wistar rats were reported instead to induce two types of activity, distinguishable both at the EEG and behavioral level 58, 59. At first intermittent bursts, that is, short (5–8 second) periods of hypersynchronous 5–6 Hz “spikes and waves”, appeared on the EEG (Figure 1E2). Concomitantly with the start and end of these intermittent bursts of “spike and waves” the animals froze with their eyes open. These intermittent bursts gradually increased in length and within 10 min evolved into a continuous hypersynchronous state at a lower frequency (4–5 Hz) (Figure 1E3). This state lasted for about 20 min during which the animal stopped moving altogether and appeared to be in a sedated state. As shown in Figure 1E3, the EEG activity in this continuous hypersynchronous state appeared to be less regular than during the intermittent bursts, with slow waves and spikes not always associated into spike‐wave complexes (SWCs). After 20 min, the intermittent bursts, and their associated behavioral output, gradually reappeared on the background of a desynchronized EEG. The authors of this study posited that the intermittent bursts of “spike and waves”, with their clear transitory interruption of directed movement, paralleled spontaneous ASs 58. No attempt was made to correlate the continuous synchronized state with human pathology. A similar progression between an intermittent synchronized EEG state and a continuous synchronized EEG state had been described in an early study 35 where equimolar doses of 5.8 mM GHB or GBL (equivalent to ~700 mg/kg GHB and ~500 mg/kg GBL) were administered to Sprague–Dawley rats. In addition, these higher doses of GHB and GBL produced a reversible burst‐suppression pattern (for 50–80 min) that was concomitant with a loss of the righting reflex (Figure 1E4). In subsequent studies of GHB‐induced activity, other fine behavioral phenotypes were also observed. During the intermittent bursts with behavioral arrest, rats were seen to display facial myoclonus and vibrissal twitching 25, features that are also present in genetic rat models of ASs 60, 61. These manifestations are said to represent the correlates of some behavioral automatisms (e.g., lip smacking, eyelid flutters, chewing) that are observed during spontaneous ASs in humans 2.

Importantly, the intermittent and continuous hypersynchronous EEG states in the rat (Figure 1E2 and E3) had a pharmacological profile strikingly similar to the one of human ASs, being blocked by drugs that are effective against spontaneous human ASs (e.g., ethosuximide and valproate) and exacerbated by drugs that are effective on convulsive seizures (e.g., carbamazepine and phenytoin) 25, 59, 62, 63. In addition, ethosuximide was ineffective in blocking the burst‐suppression pattern (Figure 1E4), suggesting that this state is distinct from GHB‐elicited ASs 64.

**Figure 3:**
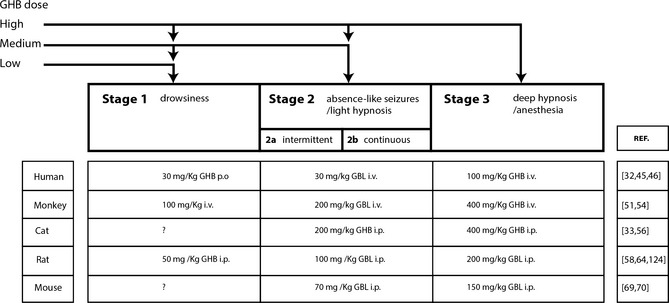
Systemic GHB administration induces three stages of activity that are distinguishable at the EEG and behavioral level. GHB (or its prodrug GBL) dose‐dependently induces marked changes in the EEG and behavior in various animal species (humans, monkeys, cats, rats, and mice). These GHB‐elicited activities can be grouped in 3 stages (top) that are reached in succession with increasing concentrations of GHB. The wearing off of the drug also follows the 3 stages but in an inverse order. The threshold dose to reach each stage is illustrated together with the route of administration. Low doses of GHB (stage 1) induce drowsiness and non‐REM sleep. Medium doses of GHB induce a peculiar phenotype that is generally thought to mimic human absence seizures and/or light hypnosis, and can be divided in two substages (a and b). During stage 2a, intermittent EEG paroxysms emerge from a background of desynchronized EEG. During stage 2b (that is reached with the same threshold dose of stage 2a), there is a light hypnotic state, characterized by changes in body posture and decrease in muscle tone, while the EEG paroxysms become continuous. At high doses of GHB, a behavioral state of deep hypnosis/anesthesia is reached (stage 3) which is accompanied by a burst‐suppression pattern in the EEG. p.o.: per os; i.v.: intravenous; i.p.: intraperitoneal; NA/?: data not available.

It is noteworthy that even though the initial study posited that only GHB‐elicited SWDs, accompanied by behavioral arrest with sudden onset and termination (Figure 1E2), could model spontaneous human ASs 58, in the subsequent literature, all of the activities evoked by GHB in the rat (with the exclusion of the burst‐suppression pattern) were said to reproduce ASs 25, 36, 64. Nonetheless the EEG and behavioral effects of GHB‐elicited ASs seem to vary among experiments and even within the same experiment (see for example Figure 3 in 65 where upon injection of 100 mg/kg GBL there is a clear slowing down of the frequency of the GHB‐elicited EEG activity with time). This is also apparent in the frequencies of GHB‐elicited SWDs and continuous hypersynchrony, which have been reported to vary across the range 3–9 Hz, and in the different terminology that researchers have used to describe these EEG manifestations (Table 1). It is currently unclear how much these differences are related to rat strain (with experiments being conducted mainly on Wistar and Sprague–Dawley rats) (Table 1) or doses of GHB/GBL.

#### Mice

Administration of low doses of GBL (50 mg/kg) in mice has generally been reported to have no effect on the EEG 66 and, in contrast to rats, failed to induce slow‐wave sleep 67, 68. Instead, a slightly larger dose (70 mg–100 mg/kg GBL) induced, after 5–10 min from the injection, a state that was generally described as EEG hypersynchrony 67, 68 or SWDs 69, 70, 71. The effect of GBL administration was generally reported to last less than in the rat, totaling 30 min to 1 h in different studies 69, 70, 72. The frequencies reported for the GHB‐elicited EEG activities are generally lower than in the rat and they vary around 3–6 Hz (Table 1). In addition, although it is never discussed directly in the literature, it appears that the so‐called SWDs in the mouse are often less regular than in the rat, and clearly discernible SWCs (where spikes and waves are phase‐locked) are seldom observed (Figure 1F2). Indeed, the most prevalent EEG activity appears to be a general shift of EEG activity to lower frequencies with occasional spikes (Figure 1F3). Higher doses of GBL (200–400 mg/kg) induced an EEG burst‐suppression pattern and, behaviorally, a loss of the righting reflex (Figure 1F4) 66, as observed in other species.

The pharmacological profile of GHB‐induced SWDs in mice has not been characterized as thoroughly as in the rat. Notably, GHB‐induced continuous hypersynchronous events and SWDs were reduced by ethosuximide (200 mg/kg), while valproate (100 mg/kg) was ineffective 70. Moreover, no data are available on the effects of carbamazepine or phenytoin on GHB‐elicited responses in mice.

### Ontogeny of GHB‐Elicited ASs

Pediatric ASs, characteristic symptom of CAE, have a peculiar developmental profile characterized by a high‐remission rate in adolescence 1. Importantly, this developmental profile is different from that of all genetic models of ASs studied, where instead ASs frequency and duration are reported to increase throughout the lifespan of the animal 16, 73. In the case of the GHB model, the ontogeny of GHB‐elicited ASs was carefully studied in the rat using 200–400 mg/kg of GBL 64. In young animals (less than postnatal day 14, P14), GHB only produced EEG slowing and a burst‐suppression pattern. Some intermediated spiking activity was evoked around P16, but full‐blown SWDs were only observed after P28 64. No differences were reported for GHB‐evoked EEG activities among adult animals of different age (P30‐P90). Thus, in the GHB model, SWDs can only be evoked in the adult brain, strengthening the similarity to other genetic models of ASs and the difference with humans.

### Classification of GHB‐Elicited Effects: Which Stage Models Human ASs?

From the description provided in the previous sections, it is clear that the effects of GHB on the EEG and behavior of different species vary dose‐dependently on a spectrum from drowsiness/sleep‐facilitating effects, to activities that resemble spontaneous human ASs, to hypnosis and anesthesia. Expanding on the classification originally introduced by Schneider for humans 32 and by Snead for rats 64, 74, here we propose a structured classification of GHB‐elicited activities into three stages, each with characteristic EEG and behavioral correlates across all animal species (Figure 3). These three stages are reached in succession and with different thresholds of GHB concentration, and the wearing off of the drug follows the same stages but in the reverse order (Figure 3).

#### Stage 1: Drowsiness/Sleep‐Facilitation

GHB produces drowsiness and a slowing down of the EEG and/or facilitates an increase in slow‐wave sleep. This stage is generally not observed in cats and mice, but is present in primates and rats (Figure 3).

#### Stage 2: Absence Seizures/Light Hypnosis

High‐amplitude slow waves and/or spikes appear in the EEG. Primates have very prominent 2–3 Hz slow/delta waves but no clear spikes in the EEG. Rats display a range of activities from SWCs (at 4–9 Hz) to slow/delta waves. Cats, in addition to 2–3 Hz SWDs and slow waves, also present intermittent trains of spikes. Generally, slow waves/SWDs start to occur intermittently in well‐isolated short periods (~5 seconds in humans and cats; ~5–8 seconds in rats) from a background of desynchronized EEG, and are invariably concomitant with a behavioral arrest and, in some species, behavioral automatisms (stage 2a) (Figure 3). Then, in all species, the EEG slow waves/SWDs become continuous, their frequency tends to slow down (humans 2–5 Hz to 2–2.5 Hz; cats 2–3 Hz to 2.5 Hz; rats 5–6 Hz to 4–5 Hz) and immobility sets in (stage 2b) (Figure 3). This continuous EEG activity is reversible and can be temporarily interrupted by sensory stimulation, which produces both a behavioral output and a desynchronized EEG. The behavior (e.g., body posture and muscle tone) observed in stage 2b is suggestive of a light hypnotic state (Figure 3).

#### Stage 3: Deep Hypnosis/Anesthesia

The slowing down of the EEG frequency progresses and, in all species, evolves into an EEG burst‐suppression pattern similar to what is observed in propofol or isoflurane anesthesia, that is, electrical silence interrupted by bursts of spikes (Figure 3) 48, 49. Behaviorally, this state is similar to deep hypnosis/anesthesia. In rodents, there is a characteristic loss of the righting reflex. Myoclonic jerks are sometimes observed in monkeys and cats.

### Does GHB Induce ASs in All Animal Species?

In all animal species examined, concentrations of GHB that reach stage 2 and stage 3 induce a behavioral phenotype that is indicative of impairment of consciousness. However, many hypnotic drugs besides GHB, such as barbiturates 33, also produce an impairment of consciousness. To model an AS, the impairment of consciousness should be sudden, transient, and devoid of convulsion. Moreover, an impairment of consciousness can be inferred by an external observer only if it has a behavioral correlate, such as a transient behavioral arrest; so, only the intermittent EEG paroxysms found in stage 2a fully meet these requirements (Table 3). In addition, the EEG paroxysm of human ASs has a unique SWD morphology. However, this human EEG morphology can vary quite substantially compared to the “textbook” representation, as often the spike component of the SWC is reduced in amplitude or appears to be buried inside the wave, in particular during the terminal phase of a SWD (Figure 1A2 and A3) 75. Nonetheless, an EEG spike component can always be observed, in at least some SWCs.

**Table 3:** Comparison of stage 2 GHB‐elicited activities and human ASs

	EEG		Behavior		Pharmacological profile	
	Stage2a	Stage2b	Stage2a	Stage2b	Stage2a	Stage 2b
Human	+	−	?	−	?	?
Monkey	?	−	?	−	?	++
Cat	+++	+	+	−	?	?
Rat	++	+	+	−	+++	+++
Mouse	+	−	?	−	+	+

+: some degree of similarity to human ASs; ++: similar to human ASs; +++: closely matching human ASs; −: different from human ASs. See main text for further details.

In the case of primates, several observations challenge the classification of stage 2 GHB‐induced activities as being similar to spontaneous human ASs. In both healthy humans and monkeys, the EEG of stage 2 GHB‐induced activities is characterized by high‐amplitude 2–3 Hz slow waves with no spike component. It is unlikely that this is due to technical limitations in the original EEG recordings 51, as the same results have been recently replicated in marmoset monkeys 54. Moreover, in an earlier human study 32, GHB was able to trigger proper SWDs but only in patients with a history of generalized seizures. This effect was temporally restricted to the transition between stage 1 and stage 2 of the GHB action (Figure 2). Upon cessation of the SWDs, the usual stage 2 GHB‐induced delta waves (devoid of EEG spikes) appeared in the EEG 32. Finally, in humans 47 and in monkeys 51, the delta waves could also be triggered by auditory or visual stimulations, a feature that is not present in typical human ASs. The practice of defining the slow/delta waves as ASs seems to be driven more from the pharmacological profile of this evoked activity than from similarities to the human condition. It is striking that stage 2 GHB‐evoked activities are abolished by ethosuximide and exacerbated by phenytoin. Nonetheless, given that (1) EEG paroxysms are devoid of a spike component, (2) the behavioral correlates are similar to hypnosis and, in humans, are accompanied by a feeling of drunkenness and reduced cognitive function (e.g., difficulty in performing mental arithmetic), and (3) the effects of antiabsence drugs against hypnotic drugs (and their EEG manifestations) have not been tested, it would be prudent to withhold judgment on whether, in primates, this stage actually models human ASs (Table 3).

In the cat, 3 Hz SW complexes, along with trains of spikes and isolated waves, are produced in stage 2. These activities bear morphological similarity to human SWDs 55, but unfortunately the sensitivity of this GHB‐elicited activity to antiabsence and anticonvulsant drugs has not been tested.

In the rat, the intermittent spike and wave bursts of stage 2a have similar EEG morphology to human SWDs. Importantly, the motor behavior of the rats is also indicative of an AS, that is, freezing for the duration of the EEG paroxysm and the resumption of previous motor behavior upon its termination. Assessing an impairment of consciousness in rats is even more challenging than it is in humans 3. Some strategies, such as comparing evoked potential during sleep/SWDs 76 or looking at ictal stimulus processing 77, have been performed in rat genetic models of ASs, but not in the GHB model. Moreover, the frequency of GHB‐elicited SWDs is higher than that in humans. However, this feature is shared by all rat pharmacological and genetic models of ASs. The reason for this phenomenon is unclear but it has been suggested that it represents intrinsic interspecies differences 78.

Stage 2b (i.e., continuous hypersynchrony) in rats was originally suggested not to model ASs. This conclusion was based on the fact that, behaviorally, the animals appear sedated (i.e., for several minutes rats are not moving), and therefore there is no evidence of interruption of a directed movement. Moreover, their posture (i.e., the animals are sitting quietly with their belly on the cage floor with a decreased muscle tone) is more suggestive of a hypnotic state than of ASs. Finally, the morphology of the EEG activity during stage 2b is different from that of the intermittent bursts of spike and waves: their frequency is lower and less regular, and the spike component is often missing. It is striking although that both stage 2a and 2b in rats have the same pharmacological profile, that is, they both respond to antiabsence drugs (Table 2). As mentioned previously, the effect of antiabsence drugs on the EEG and behaviors elicited by hypnotic drugs has never been tested, but there is some evidence that ethosuximide can reduce the duration of pentobarbital‐induced sleep in rats 79. On the basis of all these issues, therefore, we suggest that the more cautious interpretation at present is that, in rats, only stage 2a models ASs while stage 2b is more similar to sedation/hypnosis (Table 3).

In mice, stages 2a and 2b are less well defined than in rats, and no study has clearly described the transition between intermittent bursts of spike and waves and continuous hypersynchrony and their respective behavioral correlates. Isolated spike and waves in mice, with sudden onset and termination, are sometimes difficult to discern (see figure 3A and 5D in ref 69, 80, respectively). Moreover, the pharmacological characterization of GHB‐induced ASs in mice is still only partial, and some differences to the rat GHB model, such as the unresponsiveness of stage 2 GHB‐elicited activities to valproate, have not been further investigated.

In conclusion, while the GHB model in the rat has been characterized extensively, in other species, many important aspects of this characterization are still missing. The fact that in all species the activities of stage 2 are defined as ASs is misleading, and this classification should be restricted to stage 2a in rats until further investigation is carried out in other species (Table 3).

## Anatomical Substrate of GHB‐Induced ASs

In this section, we describe the mapping of GHB‐elicited activities within the brain. In particular, given that most work has been carried out in the rat, we will compare the features of the rat GHB model with other well‐characterized rat models of absence epilepsy (namely GAERS and WAG/Rij) and with human data.

### Thalamocortical Network

ASs are generated within the TC network 1, 15. Indeed, GHB‐induced ASs can be recorded simultaneously in cortex and thalamus in all the species in which this has been attempted, that is, monkey 34, cat 33, and rat 81. An extensive characterization of the neuronal substrates of GHB‐elicited seizures has been performed in the rat with both ictal and interictal local field potential (LFP) recordings in various brain areas 81 and with thalamic electrical lesions 82. Some marked differences emerged with respect to other established rat models of ASs. Firstly, GHB‐induced SWDs were recorded only in superficial layers (I–IV) of the fronto‐parietal cortex and not in deep layers (V–VI). This is surprising considering that in GAERS SWDs can be observed in LFP recordings across all cortical layers. Furthermore, the initiation site of ASs has been identified in layers V–VI of the somatosensory cortex in GAERS and WAG/Rij 83, 84. Secondly, SWDs were recorded in all thalamic nuclei, including intralaminar nuclei, with the exception of the ventrolateral nucleus 81. Conversely, in GAERS, SWDs were present in the ventrolateral nucleus, while the intralaminar nuclei were silent 61. Moreover, while lesions of the ventrobasal complex and NRT abolished ASs in GAERS 85, in the GHB model, these lesions only reduced ASs by 25% 82. In addition, lesions of the intralaminar nuclei, which are ineffective in GAERS 85, abolished GHB‐induced ASs 82.

### Hippocampus

Human fMRI studies indicate that hippocampal structures are generally silent during ASs 17, 18, 19, although to the best of our knowledge, there has been no direct electrophysiological recordings in humans to confirm this. In genetic rat models of ASs, no SWDs can be recorded in hippocampal territories 16, 60, but they are present in the hippocampus of stargazer mice 86.

In the case of the GHB model, most experimental studies have shown that an ictogenic concentration of GHB induces similar EEG activity in cortex and hippocampus. Parenteral administration of an ictogenic concentration of GHB in cats induced similar EEG activity, characterized by slow waves or SW complexes that could be recorded in cortical, thalamic, and hippocampal territories 33. The same was true for the thalamic administration of GHB in monkeys 34. In rats, the overwhelming fMRI 87 and electrophysiology 64, 88 evidence supports the presence of SWDs in the hippocampus during GHB‐elicited ASs (but see ref 81).

## Brain Targets of GHB: Molecular, Biochemical, and Pharmacological Evidence

The mechanism by which the EEG and behavioral effects of GHB are produced is only partly understood. GHB is known to bind to at least two populations of receptors in the brain: GABA_B_ receptors (GABA_B_Rs) and a putative GHB receptor (GHBR) 89, 90. Although most evidence converges to suggest that the hypnotic and ictogenic activity of GHB is mediated by its activation of GABA_B_Rs, a contribution of the putative GHB receptor is still under debate 37, 89, 90. In addition, it has been suggested that some of the effects of GHB could be due to its conversion to GABA 91 (see below). We will start by critically reviewing the evidence for the existence of a GHBR. We then discuss the *in vitro* and *in vivo* evidence that could link the activation of the putative GHBR to GHB‐induced ASs and hypnosis. We will concentrate on studies that use GABA_B_Rs/GHBR antagonists and GHB at doses that are relevant for the generation of ASs. Moreover, we will focus on brain areas that are known to be important for the expression of ASs in the GHB model, that is, the TC network, but also on the hippocampus where GHB‐elicited SWDs have been recorded in rats (as discussed above). For a summary of GHB‐elicited actions in other brain areas, readers are referred to relevant reviews 89, 92.

### Molecular Identity of the Putative GHBR

The existence of a GHBR was originally suggested by the presence of high affinity (nM) binding sites for GHB in the rat brain 93. This specific binding starts to be observed at postnatal day 15–18 and reaches full expression at 3–4 weeks postnatally; binding is most intense in the hippocampus, cortex, thalamus, and amygdala, with the cerebellum showing very low binding levels 94. These high affinity sites do not correspond to GABA_B_Rs, to which GHB binds only with low affinity (in the *μ*M range) 31, 95. This is demonstrated by the fact that GHB binding on the high affinity binding sites cannot be displaced by GABA or baclofen 31 and that the high affinity binding sites are spared in GABA_B_ knockout mice 96, 97. The molecular identity of the GHBR(s) is at present unknown and has been the subject of controversy for the last 30 years. The high affinity GHB binding sites are increased during GHB‐induced ASs 81 and in the thalamus of adult GAERS rats compared to nonepileptic controls 98, although it is not clear whether this is a cause or a consequence of ASs.

Initially, the presence of the GHBR was studied using various compounds able to displace GHB binding on the high affinity GHB binding sites. These compounds would be, in principle, useful tools to understand the cellular effects of activation/inhibition of the putative GHBR, but unfortunately their intrinsic properties are unclear. The best‐characterized GHBR ligand is NCS‐382 which, as well as displacing GHB in binding studies, was shown to antagonize GABA_B_‐independent GHB effects at least in a limited number of studies (described in the following sections). Several GHB analogs have also been produced (reviewed in 99, 100), but there is very little evidence, *in vivo* or *in vitro,* that these compounds activate the putative GHBR (i.e., that they mimick GABA_B_ ‐independent effects of GHB).

From this analysis, it is clear that the controversy on the role of the GHBR is unlikely to be resolved until the molecular identity of the protein comprising the high affinity binding site of GHB is fully isolated. In that respect, there have been two reports of the identification of a high affinity molecular target of GHB. In 2003, Andriamampandry et al. 101 described the cloning of a rat GHB receptor. Nonetheless, this putative GHB receptor does not bind NCS‐382 and its expression only partially overlaps with the GHB high affinity binding site 101. For instance, it is highly expressed in the cerebellum, an area with the lowest expression of GHB high affinity binding sites 94.

In 2012, a study by Absalom et al. 102 reported that extrasynaptic GABA_A_ receptors (GABA_A_Rs), and in particular the *α*4*β*1*δ* GABA_A_R, could represent a population of the elusive GHB receptors. Indeed, *α*4*β*1*δ* receptors expressed in xenopus oocytes were activated by nM concentrations of GHB. Using autoradiography, GHB, and gabazine, but not GABA, were shown to displace the novel GHBR ligand [^125^I]BnOPh‐GHB. Furthermore, it was reported that NCS‐382 binding was reduced by ~40% in *α*4 knockout mice 102. This discovery could be of great importance because extrasynaptic GABA_A_Rs are responsible for the tonic GABA_A_ current, which has been shown to be increased in the thalamus in various models of ASs 103, and to influence sleep and anesthesia 104. Nonetheless, the relevance of these observations to native brain tissue has recently been called into question. In fact, it has been demonstrated that, in thalamus, hippocampus, and cerebellum, three regions where *α*4*β*1*δ* GABA_A_ receptors are highly expressed, *μ*M concentrations of GHB were not able to evoke any activity on extrasynaptic GABA_A_Rs 105. In addition, mM concentrations of GHB could only evoke activity dependent on GABA_B_Rs. All these observations, together with the fact that the binding of NCS‐382 to *α*4*β*1*δ* receptors was not tested, means that the identity of the high affinity binding site of GHB is still uncertain.

## Putative GHBR‐Mediated Effects: ASs, Cellular Excitability, and Synaptic Potentials

Controversy also still surrounds the effect of systemic administration of the GHB antagonist NCS‐382 on ASs. It was initially reported that the compound blocked various GHB‐induced effects such as cataplexy, hypolocomotion, and ASs 106, 107, but more recent studies have reported a lack of effects 108, 109, 110 or even an aggravation of GHB‐induced activities 100, 111. In addition, high doses of NCS‐382 were able to drastically reduce SWDs induced by PTZ 112 and spontaneous ASs in GAERS 107 and lethargic mice 113, suggesting that its effects are not specific to the GHB model. Finally, putative agonists for the GHBR (e.g., trans‐hydroxycrotonic acid (THCA), which displaces GHB from its high affinity binding sites, but does not bind to GABA_B_Rs 93), did not induce ASs in naïve animals or exacerbate seizures in GAERS 100, 114.

As far as potential GHBR‐mediated effects of GHB are concerned, an *in vivo* study in anesthetized mice showed that NCS‐382 blocked the increase in long‐term potentiation in the hippocampus elicited by systemic GBL (50 mg/kg) 113. Unfortunately, the effect of GABA_B_R antagonists, that blocked a similar increase in long‐term potentiation induced by baclofen, was not tested against the GBL action 113. Another *in vivo* study in anesthetized rats reported that systemic GHB (5–10 mg/kg) first decreased and then increased the firing rate of unidentified cortical layer III–VI neurons, with the latter effect being blocked by NCS‐382 115. The results of these studies should be interpreted with caution as effects resulting from systemic administration of GHB (and NCS‐382) may involve actions that are not direct on the recorded neurons or the brain region under investigation.

A direct action on hippocampal neurons was shown in *in vitro* studies on CA1 pyramidal neurons, where GHB reduced the amplitude of both EPSPs (at 600 *μ*M) and IPSPs (at 100–1200 *μ*M) 116, 117. Importantly, the effects of GHB were antagonized by NCS‐382 but were not affected by GABA_B_R antagonists. Another NCS‐382‐dependent action in the hippocampus is the increase in glutamate levels observed after local microdialysis application of 100–500 nM of GHB 118, 119. Interestingly, an increase in glutamate levels was also obtained with the putative GHBR agonist THCA 118. In contrast, microdialysis of 1 mM GHB elicited a decrease in hippocampal glutamate levels that was only partially blocked by NCS‐382 but fully antagonized by GABA_B_R antagonists 119.

## GABA_B_Rs as Targets of GHB

GHB is a weak agonist at GABA_B_Rs: it displaces binding of the GABA_B_ agonist baclofen with a Kd in the range of 30–500 *μ*M 31, 95 and activates heterologous GABA_B_Rs with an EC50 in the low mM range 120. This is particularly significant considering the concentration of endogenous GHB in the brain is 1–4 *μ*M 121, 122 and that the threshold brain concentration of GHB that correlates with the onset of an AS phenotype is 240 *μ*M 39. Furthermore, the hypnotic/anesthetic concentration of GHB in the brain (measured when animals regain their righting reflex after bolus i.v. administration of GHB) is 400 *μ*M 123. Therefore, ictogenic and anesthetic/hypnotic concentrations of GHB are compatible with activation of GABA_B_Rs.

### Global Blockade of GABA_B_Rs: Effects on ASs and Behavior

Most, if not all, behavioral effects, including ASs 112, 124 and hypnosis/anesthesia 125, induced by exogenous GHB administration *in vivo,* can be blocked by GABA_B_R antagonists (reviewed in 125), whereas the sensitivity of the putative sleep‐inducing effect of low doses of GHB (e.g., 50 mg/kg in the rat, 57, 58) to these drugs has not been tested. Recently, experiments in GABA_B_R knockout mice have provided compelling evidence that the majority of the effects induced by GHB are dependent on the presence of these receptors. At a range of doses that encompass the sedative, pro‐epileptic and anesthetic concentrations of exogenously administered GHB (50–300 mg/kg), no effects were observed in these knockout mice 68, 96.

Finally, the potent and selective GABA_B_ agonist baclofen induces similar EEG and behavioral effects to GHB, both in terms of an AS‐like phenotype 112 and anesthetic action 126. Nonetheless, the effects of systemic baclofen have not been fully characterized as an absence epilepsy model (e.g., pharmacological profile, anatomical substrates of SWDs, etc.) so the association of baclofen to an AS phenotype remains tentative.

### GHB Activation of GABA_B_Rs in the Thalamus

*In vitro* studies in slice preparations of the cat and rat thalamus have demonstrated multiple postsynaptic effects of GHB on TC neurons. GHB elicits a membrane hyperpolarization on TC cells; this effect is dose‐dependent starting from 100 *μ*M (the lowest concentration tested) to 3 mM (the plateau of the effect) 127. The effect was mediated by the opening of potassium channels and, at the doses tested (400 *μ*M–1 mM), was blocked by a GABA_B_R antagonist. Recent work has also produced evidence of another important thalamic postsynaptic effect of GHB: an increase in tonic GABA_A_ inhibition 103 (see below). This effect is postsynaptic and is mediated by GABA_B_Rs via a G‐protein‐dependent pathway that probably results in dephosphorylation of extrasynaptic GABA_A_Rs 128.

Presynaptic effects induced by GHB on TC cells have also been described: GHB reduced the amplitude of sensory and corticothalamic EPSPs 129, 130. The minimum concentrations of GHB to induce these reductions were 100 *μ*M and 250 *μ*M, respectively. Interestingly, GABA IPSPs originating from the NRT were only reduced by GHB concentrations ≥500 *μ*M 130. All these effects were blocked by GABA_B_ antagonists, while NCS‐382 was either ineffective or had a tendency to potentiate the effects of GHB 129, 130.

Finally, an *in vivo* study looked at the effects of GHB, injected systemically or in the ventrobasal thalamus by reverse microdialysis, on basal and K^+^‐evoked glutamate and GABA levels 65. Starting from a concentration of 250 *μ*M, GHB reduced basal GABA levels, leaving basal glutamate levels unchanged; both GABA and glutamate K^+^‐evoked levels were instead reduced. A similar effect on levels of these neurotransmitters in the ventrobasal thalamus was observed during GHB‐induced ASs. These effects were fully blocked by systemic application of GABA_B_ antagonists but only partially antagonized by NCS‐382 65. Nonetheless, the significance of changes of basal and K^+^‐evoked levels of neurotransmitters for local network activities are difficult to predict in the absence of electrophysiological data on neuronal firing dynamics.

### GHB Activation of GABA_B_Rs in the Cortex

An *in vivo* study 131 in anesthetized rats showed that systemic administration of GHB at 100 mg/kg had no effect or a tendency to increase the firing rate of unidentified pyramidal cells, whereas 300 mg/kg produced decreases in the firing rate. In another investigation 115, systemic injection of GHB (160–320 mg/kg) decreased the firing rate followed by a rebound increase. These effects were not sensitive to NCS‐382, but GABA_B_ antagonists were not tested. The paucity of *in vivo* electrophysiological studies does not allow us to draw a clear conclusion on the effects on GHB on the cortex *in vivo*. In addition, as mentioned earlier, the possibility remains that the effects observed after systemic administration of GHB are indirect.

An *in vivo* dialysis study investigated basal and K^+^‐evoked release of GABA and glutamate in the superficial layers of the cortex 132. While glutamate concentration was unchanged, there was a clear reduction of both basal and K^+^‐evoked GABA levels. This effect was blocked both with GABA_B_ antagonists and with NCS‐382. It is worth noting that the effects on GABA release ended within 70 min, while ASs persisted for 2 h. It is therefore unclear if these effects simply co‐occur with ASs after GBL application or if they have a mechanistic role in their expression.

Experiments in slice preparations from the mouse frontal cortex have shown that, similar to the thalamus, GHB induces a hyperpolarization both of pyramidal cells and putative interneurons in layer II/III, with the difference that the threshold to obtain this effect was reported to be in the mM range 133. In addition, GHB (1 mM) caused a depression in both amplitude and frequency of miniature EPSPs and IPSPs via a presynaptic mechanism. A more recent study 134 in the rat prefrontal cortex demonstrated that 300 *μ*M of GHB was sufficient to reduce the amplitude of NMDA‐EPSPs in layer II/III pyramidal cells. AMPA EPSPs and IPSPs were only depressed at a concentration of 1 mM. All the aforementioned effects, either in mouse or rat, were fully antagonized by GABA_B_ antagonists, while NCS‐382 was ineffective against the GHB‐elicited depression of postsynaptic potentials 133, 134.

### GHB Activation of GABA_B_Rs in the Hippocampus

As discussed above, the hippocampus, the area with the highest expression of GHB high affinity binding sites, is also the only area where it is possible to observe clear electrophysiological effects of GHB that are antagonized by NCS‐382, but not by GABA_B_ antagonists. Nonetheless, in the hippocampus, GHB can also elicit GABA_B_ specific effects. An early study reported that, similar to cortex and thalamus, GHB produces a GABA_B_‐dependent hyperpolarization of CA1 pyramidal neurons 135. In contrast to the work of Berton et al. 116(described above), 1 mM GHB decreased EPSPs and IPSPs via GABA_B_R‐mediated action 136.

## GHB Effects on Astrocytes: Actions on GHBR and GABA_B_Rs

Although astrocytes were once considered to have only a structural function in the brain, they have now been shown to play a substantial role in synaptic physiology 137, 138. Moreover, a role for astrocytes in genetic models of ASs is being increasingly recognized 139. Less is known about the effects of GHB on astrocytes of the TC network. A recent study 140, however, has shown that GHB dose‐dependently induced a transient increase of intracellular Ca^2+^ in astrocytes of the rat ventrobasal thalamus, with an ED50 of 1.6 mM and a minimal effective concentration of 250 *μ*M. These GHB‐elicited astrocytic Ca^2+^ transients were abolished by GABA_B_R antagonists and absent in GABA_B_R knockout mice. Similar results were obtained with the GABA_B_ agonist baclofen. NCS‐382 was ineffective in blocking the baclofen‐evoked Ca^2+^ transients but drastically reduced those elicited by GHB. These results suggest that activation of GABA_B_Rs is necessary to produce Ca^2+^ transients in thalamic astrocytes 140.

## Metabolic Conversion of GHB to GABA

The possibility that the effects of exogenously administered GHB may be due to its metabolic conversion to GABA (which occurs via a cytosolic GHB dehydrogenase 31, 141) has been debated for many years 91, 95. Several lines of evidence, however, strongly suggest that the amount of GHB metabolized to GABA is not substantial, especially at GHB concentrations relevant for its pro‐epileptic/hypnotic actions. Firstly, the electrophysiological effects of GHB are blocked by GABA_B_ antagonists but not by GABA_A_ antagonists. Yet, if GHB were converted to GABA, an effect on both receptors would be expected. Secondly, in GABA_B_ knockout mice, high doses of GHB (>1000 mg/kg) do not induce phenotypes that would be expected from an increased GABA_A_R activation (as discussed above). Thirdly, ethosuximide and valproate at concentrations that inhibit the GHB dehydrogenase 141 had no effect on GHB effects mediated by GABA_B_Rs 95, 134, 142. In conclusion, the current evidence strongly supports the view that the ability of exogenously administered GHB to induce ASs does not depend on its metabolic conversion to GABA.

## Mechanism of GHB‐Induced ASs in the Rat

In this section, we describe potential mechanisms for the generation of GHB‐elicited ASs in rats. As discussed above, we define GHB‐induced ASs only the EEG and behavioral activity that is characteristic of stage 2a in this species. Moreover, it is worth noting that, compared to the thorough mechanistic description of spontaneous ASs in genetic models of absence epilepsy, there is a paucity of studies exploring the mechanisms of the GHB‐elicited ASs. In particular, in GAERS and WAG/Rij 83, 84, as well as in humans 18, 19, there is evidence of a cortical initiation site. Whether this is the case in the GHB model remains to be ascertained, although there is some evidence that, within the cortex, seizures are initiated in the primary somatosensory cortex 143. In addition, in GAERS, intracellular or extracellular recordings from thalamic and cortical neurons have provided a clear view of their firing dynamics during ASs, *in vivo* 83, 144, 145, whereas a similar description is not available for GHB‐elicited ASs.

In view of the *in vitro* and *in vivo* evidence presented in the previous sections, it seems safe to assume that GHB, acting as a weak agonist on GABA_B_Rs, is responsible for the electrophysiological effects observed in the TC network. At brain concentrations relevant for the expression of ASs (i.e., 240 *μ*M), GHB induces a postsynaptic hyperpolarization on corticothalamic and TC neurons and, both in the ventrobasal thalamus and in the frontal cortex, presynaptically depresses EPSPs, therefore favoring phasic inhibition over phasic excitation.

In addition, several lines of evidence suggest that GHB activation of GABA_B_Rs in the thalamus is involved in the generation of SWDs. Intrathalamic and systemic administration of GHB produces similar EEG and behavioral activities in both monkeys 34 and rats 39, as does intrathalamic administration of baclofen 146. Another indication of the role of the thalamus in the expression of GHB‐elicited ASs has come from work on thalamic tonic GABA_A_ inhibition. This type of inhibition is increased in several animal models of absence epilepsy compared to nonepileptic controls and this has been shown to be causally linked to the expression of the disease 103, 147. GHB (300 *μ*M) produces a ~30% increase in tonic GABA_A_ current 103, an effect that is mediated by a postsynaptic cross‐talk between GABA_B_Rs and GABA_A_Rs 128 (as discussed above).

Therefore, GHB acts on TC neurons with at least three independent mechanisms that could facilitate/generate ASs: presynaptically by an indirect increase in phasic GABA_A_ inhibition, and postsynaptically by membrane hyperpolarization and an increase in GABA_A_ tonic inhibition. Notwithstanding this evidence, a contribution of the cortex in the generation of GHB‐induced ASs cannot be ruled out. If the identification of a cortical initiation site in GHB‐elicited ASs were to be confirmed, it could open new avenues of investigation on the cortical contribution to the generation of GHB‐induced ASs. Finally, the lack of *in vivo* recordings of cortical and thalamic neurons during GHB‐elicited ASs does not allow us, at present, to predict how the aforementioned *in vitro* mechanisms converge to evoke an AS.

## Voltage‐ and Neurotransmitter‐Gated Channels in the GHB Model

The GHB model has been used to understand the contribution of various voltage‐ and neurotransmitter‐gated channels to the expression of ASs. Broadly speaking, these experiments can be divided into two main sets: (1) pharmacological manipulations, with intracerebral or systemic drug application in rats, and (2) experiments using the GHB model in knockout mice. We will discuss these experiments in separate sections to account for the aforementioned problems with the GHB model in mice.

### Pharmacological Manipulations in the Rat

Systemic administration of both NMDA agonists and antagonists blocked the expression of GHB‐elicited SWDs but also induced a burst‐suppression pattern 148. Bilateral infusion of NMDA in thalamic nuclei and in the NRT suppressed the expression of GHB‐elicited SWDs 149. As far as the GABAergic system is concerned, systemic administration of the GABA_A_ agonist muscimol 150, or of weak GABA_A_ antagonists (PTZ, penicillin), induced an increase in GHB‐elicited ASs 36. Systemic and intrathalamic administration of steroid modulators of GABA_A_ receptors (alphaxalone, ganaxolone, tetrahydrodeoxycorticosterone) exacerbated GHB‐elicited SWDs 151, 152. Importantly, caution should be exerted in interpreting the results of all these studies, as in all of them, both stage 2a and stage 2b of GHB‐elicited activities (i.e., ASs and hypnosis) were analyzed together.

### Genetic Manipulations in the Mouse

In the last decade, different mouse knockouts have been used to investigate the role of specific genes in the expression of GHB‐elicited ASs and may, ultimately, provide some indication of the genetic abnormalities underlying these nonconvulsive seizures in humans. For example, metabotropic glutamate receptor 4 knockout mice were resistant to ASs induced by GABA_A_ antagonists (PTZ, bicuculline), while GBL (100 mg/kg) and baclofen elicited ASs as in wild‐type littermates 71. Moreover, knockout mice for the AMPA subunit GluR2 had a lower latency of onset, and a decreased cumulative duration of GHB‐elicited ASs (GBL 100 mg/kg) compared to their wild‐type littermates 153.

T‐type Ca^2+^ channels have long been thought to play a role in ASs 154. Mice lacking the *α*1G T‐Type Ca^2+^ channel gene, and which therefore do not express functional Cav3.1 channels, were resistant to GHB‐elicited seizures (GBL i.p. 70 mg/kg), while the susceptibility of these knockout mice to GABA_A_ antagonist‐elicited ASs was unchanged 69. Ca_V_2.3 channel (R‐type) knockout mice displayed a reduced cumulative time spent in seizure and reduced seizure length after systemic application of GBL (70 mg/kg) 155.

Mice lacking the GABA_A_R *α*3 subunit had a reduced length and amplitude of GHB‐elicited ASs (GBL 100 mg/kg) compared to their wild‐type littermates, and a similar result was found for systemic PTZ‐induced ASs 80. In contrast, mice lacking the δ subunit of the GABA_A_R were resistant to GHB‐induced ASs (50 mg/kg GBL) 103.

Of course, caution is needed in drawing mechanistic conclusions from these experiments as the global deletion of a gene in the CNS causes the loss of a protein that is often expressed in multiple brain areas which are part of, or project to, the TC network that is ultimately responsible for AS generation. In addition, GHB does not bind, nor has any direct electrophysiological effects on, either iono/metabotropic glutamate receptors, voltage‐gated Ca^2+^ channels or GABA_A_Rs (except at extrasynaptic GABA_A_Rs, as discussed above). Therefore, the results of all the studies presented above inform us about proteins that are necessary for the expression of GHB‐elicited ASs at the network level, but not about direct targets of the molecule. Moreover, given that these experiments have been performed in mice, where the GHB model is only partially characterized, it is difficult to predict whether these findings on knockout mice apply to the mechanism by which GHB induces ASs, hypnosis or both. Thus, crossing the aforementioned knockout mice with genetic mouse models of absence epilepsy (e.g., stargazer, tottering or lethargic mice) could be a useful tool to test independently the contribution of individual genes to the expression of ASs. Finally, it is noteworthy that the knockout of some of these genes has no effect on GABA_A_ antagonist‐induced SWDs while abolishing GHB‐induced ASs 69 or vice versa 71. Although the reasons of this difference are at present unclear, this finding clearly points to multiple independent mechanisms underlying the generation of ASs induced by different pharmacological agents, as previously suggested 154, 156, and, importantly, provides support to the hypothesis that different and independent genetic abnormalities may underlie human ASs in various patient cohorts 1.

## Summary

To help the reader, here we summarize the main points arising from our analysis of the GHB model and highlight the most pressing questions that remain unanswered.

### Only GHB‐Elicited Stage 2a Models ASs in the Rat

Both in rats and in mice, where the GHB model has been extensively used, researchers tend to identify all activities evoked by a nonanesthetic dose of GHB as ASs. We argue that this classification is incorrect as it lumps together activities that resemble ASs with others that represent a sedated/hypnotic state. Stage 2a in rats (Table 3), where the animals display SWDs accompanied by behavioral arrest, has been thoroughly characterized, in terms of EEG and behavior, and has the same face validity as genetic AS models. Stage 2b, where the EEG paroxysm becomes continuous and is accompanied by changes in body posture and muscle tone that more closely resemble hypnosis than ASs, should be considered as a distinct phenomenon. This classification has important consequences for the practical use of the GHB model. For instance, in rats, stage 2a has much shorter cumulative duration than stage 2b. Upon injection of 200 mg/kg GHB, stage 2a has 5–8 second long SWDs that occur intermittently over 10 min, whereas stage 2b has continuous hypersynchronous EEG and hypnosis lasting ~20 min, 58. Therefore, the results of many studies are skewed toward the hypnotic effects of GHB rather than GHB‐elicited ASs. Finally, the practice of considering stage 2a and stage 2b together seems to be driven mainly by pharmacological considerations. Undoubtedly, the unique pharmacological profile of ASs should contribute toward defining a model of ASs (as it defines the model predictive validity) (Table 2), but cannot be used as a substitute for EEG and behavior that resembles, that is, has face validity for, ASs. In this respect, a direct comparison, in the same animals, of the effect of ethosuximide, valproate, carbamazepine, and phenytoin on the EEG and behavior of GHB stage 2a and 2b versus sedation, hypnosis, and natural slow‐wave sleep could be of great significance.

### The GHB Model in the Mouse

The GHB model in the mouse is not as well characterized as in the rat. In particular, (1) the EEG of stage 2a appears to vary greatly between studies (Figure 1F2), and an EEG morphology with clear spikes is rarely observed; (2) as mentioned previously for the rat, in a model of ASs, SWDs should be accompanied by behavioral arrest: a prolonged lack of movements accompanied by a decrease of muscle tone and change in body posture 67, 68, 157, as observed in stage 2b in mice, is more reminiscent of an hypnotic state than ASs; (3) the pharmacological characterization of the GHB model in mice is incomplete. It is only by resolving these issues that we will be able to accept, with confidence, the GHB model in mice. Nevertheless, administration of GHB to mice has opened interesting questions about the different mechanisms that underlie ASs induced by either GABA_B_ agonists (i.e., GHB, baclofen) and GABA_A_ antagonists (i.e., PTZ, bicuculline), which are worth investigating further.

### EEG Seizure Properties in the GHB Model

While it has been suggested that ASs in the GHB model can be quantified in the same way as in polygenic rat models 25, 158, it appears from the literature that the information available for various seizure parameters, for example, average seizure length, morphology of SWDs, dominant frequency, etc) (Table 1) is scarce or contradictory. This may be, in part, due to a lack of consensus regarding which of the activities evoked by exogenous, GHB administration represents an AS 36, 58, 64. Moreover, current seizure detection methods for the GHB model have not been shown to be able to discriminate between sleep, hypnosis and ASs 69, 80.

### Molecular Targets of GHB

It is now well accepted that most, if not all, effects of exogenously administered GHB depend on its agonist action on GABA_B_Rs. Activation of the putative GHBR does not underlie the pro‐epileptic and hypnotic effects of GHB.

### Role of the Hippocampus in the GHB Model

It is remarkable that GHB‐induced ASs can be recorded in the hippocampus as limbic structures are not normally associated with typical ASs 1, 20. Importantly, the hippocampus is the brain region with the highest expression of GHB high affinity binding sites and where some electrophysiological evidence exists of GABA_B_R‐independent GHB effects. Nonetheless, mice overexpressing the GABA_B_R1a subtype have an atypical AS phenotype which involves, along with other neurological symptoms, the presence of SWDs that are not necessarily associated with behavioral arrest and that can be recorded in hippocampal territories 159. Further studies are needed to understand the role of the hippocampus during GHB‐induced ASs and the role that activating hippocampal GABA_B_Rs/GHBR plays in the genesis of typical and atypical ASs.

### Role of Thalamus and Cortex in the Expression of GHB‐Elicited ASs

Many converging studies suggest that the thalamus is a key area in the generation of GHB‐elicited ASs. Various pre‐ and postsynaptic mechanisms have been described in TC neurons. In particular, GHB increases the tonic GABA_A_ current, a key factor in the generation of ASs 103. This effect, produced by a cross‐talk between GABA_B_ and extrasynaptic GABA_A_ receptors 128, has emerged as a new player in the thalamic contribution to the generation of ASs. As far as the cortex is concerned, in contrast to current notions, on how ASs are generated and propagated 1, 83, deep layers of the cortex are silent during GHB‐induced ASs in the rat 81. This issue needs to be further investigated *in vivo*, using simultaneous recordings of LFP and unit activity across cortical layers.

## Conclusions

Although the link between GHB and ASs dates back to more than 50 years 32, 33, the description of the cellular, molecular, and behavioral aspects of the GHB model has fallen behind compared to our understanding of the genetic models of ASs. Nonetheless, pharmacological models of ASs such as the GHB model are still important: the mechanistic knowledge of how targeting a single receptor can acutely bring about an ASs represent great advantage with respect to polygenic rat models of ASs, where the underlying genetic abnormalities are unknown, and monogenic mouse models of ASs, which have other comorbidities and where developmental changes to network excitability cannot be easily tracked. Human ASs are a complex phenomenon which accompanies a wide range of epilepsies (in pediatric, juvenile, and adult patient cohorts). To further understand the pathophysiology of ASs, there is a clear need to have different tools, and thus answering the issues highlighted in this review is necessary to make the GHB model one of such indispensable tools.

## Conflict of Interest

The authors declare no conflict of interest.
